# Sample preparation of bone tissue for MALDI-MSI for forensic and (pre)clinical applications

**DOI:** 10.1007/s00216-020-02920-1

**Published:** 2020-09-15

**Authors:** Michiel Vandenbosch, Sylvia P. Nauta, Anastasiya Svirkova, Martijn Poeze, Ron M.A. Heeren, Tiffany Porta Siegel, Eva Cuypers, Martina Marchetti-Deschmann

**Affiliations:** 1 KU Leuven Toxicology and Pharmacology, Campus Gasthuisberg, Onderwijs en Navorsing 2, Herestraat 49, PO Box 922, 3000 Leuven, Belgium; 2 Maastricht MultiModal Molecular Imaging (M4I) Institute, Division of Imaging Mass Spectrometry, Maastricht University, Universiteitssingel 50, 6229 ER Maastricht, Netherlands; 3 Department of Orthopedic Surgery and Trauma Surgery, Maastricht University Medical Center, P. Debyelaan 25, 6229 HX Maastricht, Netherlands; 4 Institute of Chemical Technologies and Analytics, Division of Imaging and Instrumental Analytical Chemistry, TU Wien (Vienna University of Technology), Getreidemarkt 9/164, 1060 Vienna, Austria; 5 Division of Trauma Surgery, Department of Surgery, Maastricht University Medical Center, P. Debyelaan 25, 6229 HX Maastricht, Netherlands; 6 NUTRIM, School for Nutrition and Translational Research in Metabolism, Maastricht University, Universiteitssingel 40, 6229 ER Maastricht, Netherlands

**Keywords:** Bone tissue, Sample preparation, MALDI, Mass spectrometry imaging

## Abstract

In the past decades, matrix-assisted laser desorption/ionization mass spectrometry imaging (MALDI-MSI) has been applied to a broad range of biological samples, e.g., forensics and preclinical samples. The use of MALDI-MSI for the analysis of bone tissue has been limited due to the insulating properties of the material but more importantly the absence of a proper sample preparation protocol for undecalcified bone tissue. Undecalcified sections are preferred to retain sample integrity as much as possible or to study the tissue-bone bio interface in particular. Here, we optimized the sample preparation protocol of undecalcified bone samples, aimed at both targeted and untargeted applications for forensic and preclinical applications, respectively. Different concentrations of gelatin and carboxymethyl cellulose (CMC) were tested as embedding materials. The composition of 20% gelatin and 7.5% CMC showed to support the tissue best while sectioning. Bone tissue has to be sectioned with a tungsten carbide knife in a longitudinal fashion, while the sections need to be supported with double-sided tapes to maintain the morphology of the tissue. The developed sectioning method was shown to be applicable on rat and mouse as well as human bone samples. Targeted (methadone and EDDP) as well as untargeted (unknown lipids) detection was demonstrated. DHB proved to be the most suitable matrix for the detection of methadone and EDDP in positive ion mode. The limit of detection (LOD) is estimated to approximately 50 pg/spot on bone tissue. The protocol was successfully applied to detect the presence of methadone and EDDP in a dosed rat femur and a dosed human clavicle. The best matrices for the untargeted detection of unknown lipids in mouse hind legs in positive ion mode were CHCA and DHB based on the number of tissue-specific peaks and signal-to-noise ratios. The developed and optimized sample preparation method, applicable on animal and human bones, opens the door for future forensic and (pre)clinical investigations.

**Electronic supplementary material:**

The online version of this article (10.1007/s00216-020-02920-1) contains supplementary material, which is available to authorized users.

## Introduction

The range of applications of mass spectrometry imaging (MSI) has grown exponentially over the last decades [1, 2]. A main advantage of MSI is the possibility to detect a wide range of molecules and visualize their distributions without the need for targeted labels while maintaining sample integrity [1]. The development of matrix-assisted laser desorption/ionization (MALDI)-MSI has contributed to a broader application field for MSI, due to the soft ionization of molecules and the broader molecular weight range than previous ionization techniques, for example, secondary ionization mass spectrometry (SIMS) [1]. MSI can be applied to a wide variety of sample types, including tissue samples. As a consequence, MSI has been used more and more in (pre)clinical research, including tissue classification, studying treatment efficacy, and biomarker discovery [3]. Next to this, MALDI-MSI is used to study in situ drug, metabolite, and lipid distributions [3, 4]. The sample preparation workflow for MALDI-MSI is highly critical for the quality and reliability of the MSI measurements. On one side, the measurements depend on the analytes of interest. Small molecules can be masked by endogenous molecules or signals from the selected matrix [5]. On the other side, the quality and reliability depend on the complexity of the tissue of interest. The sample preparation will affect, among others, sample integrity, the ionization efficiency of molecules, and local ion suppression due to salts and endogenous compounds in the tissue.

For calcified tissues, like bone, decalcification is often performed to allow for easier sectioning and to reduce insulating properties of the sample, but more over to remove the unwanted background signal in MALDI-MSI caused by the mineral structure of these tissues [6]. These procedures, however, pose a high risk of contamination and analyte delocalization [7]. Despite the wide range of tissues that have been studied using MALDI-MSI, there are only a few studies that apply this technique on skeletal tissue [7–11]. This can be explained by the absence of proper section preparation protocols for undecalcified bone tissue. Undecalcified bone tissue is preferred to avoid solvent-induced analyte delocalization, which better preserves the in vivo distribution. The lack of existing sample preparation protocols can be explained by the complex morphology of bone tissue [7, 9, 11]. The combination of hard (bone), stiff (cartilage, tendons, skeletal muscle), and rather soft tissue (bone marrow, surrounding periosteum, smooth muscle) in the skeletal tissue makes sectioning a challenge [7, 11]. Bone sections have the tendency to curl or fall apart [11]. Another difficulty lies in the creation of homogenous tissue sections with MALDI-MSI-compatible techniques [1]. Previous methods for sectioning skeletal tissue have been shown to be successful in preparing bone sections [8]. However, they often involve decalcification and/or dehydration methods, which consist of washing steps using an acid [7, 11]. In 2014, Hirano et al. reported an application of MALDI-MSI on tooth cryosections, which were prepared using adhesive film without any pretreatment of the tissue [6]. Nevertheless, teeth have a different texture than bone tissue. In 2018, Svirkova et al. adapted Hirano’s method to make cross-sectional sections of chicken digits, which only contain small and thin bone pieces [10]. These and the different studies provided in Electronic Supplementary Material (ESM) Table S1 show that it is possible to cut samples containing bone and surrounding soft tissue, although each of the methods is different. Here, we aim to optimize the sample preparation and analytical workflow of undecalcified longitudinal bone sections for MALDI-MSI without any pretreatment. We will focus on the evaluation of the potential of MALDI-MSI for targeted and untargeted applications, namely the detection of small exogenous molecules (i.e., drugs of abuse) for the forensic field and endogenous ones (i.e., lipids) for the (pre)clinical field in longitudinal bone tissue sections.

During the last decade, the interest in the usage of skeletal tissue as an alternative specimen in forensic toxicology has seen a revival [12–14]. It is shown that the bone tissue acts as a depot for certain drugs [12]. However, the analysis of bone tissue is still in its infancy. Nowadays, the gold standard for analysis of skeletal tissue is the usage of liquid or gas chromatography MS [15]. Although it is of great value, this technique requires sample clean-up and extractions steps. MALDI-MSI could prove a more time efficient alternative for the detection of certain drugs. Therefore, in this study, the targeted detection of methadone and its metabolite 2-ethylidene-1,5-dimethyl-3,3-diphenylpyrrolidine (EDDP) in skeletal tissue will be studied using MALDI-MSI.

In the clinical field, the application of MALDI-MSI related to skeletal diseases can have a great potential, as the current molecular understanding of the underlying processes of, for example, bone fracture healing and non-union development is very limited [16–18]. The application of MALDI-MSI on undecalcified bone tissue can be of additional value for improved understanding of molecular pathways involved in different bone diseases. The untargeted application will be focused on different lipid classes, because lipids have a regulatory function in healthy bone and repair processes and are present in bone marrow as well as in bone [7, 19]. In this study, a method for untargeted detection of a broad range of lipid classes with MALDI-MSI in bone tissue will be optimized by comparing the efficiency of different matrices.

## Materials and methods

### Chemicals and materials

Analytical reference standards of EDDP (1 mg/mL) and methadone (1 mg/mL) were purchased from Cerilliant (Round Rock, TX, USA). Aqueous standard stocks of different concentrations were prepared by mixing reference standards. All standard solutions were stored at − 20 °C.

Acetone, ACN, methanol, isopropanol, and water were purchased from Biosolve BV (Valkenswaard, The Netherlands) with HPLC grade (acetone) or ULC/MS–CC/SFC grade (other solvents). Red phosphorus was obtained from Sigma-Aldrich (St. Louis, MO, USA).

Tragacanth, carboxymethyl cellulose (CMC) sodium salt, and gelatin were purchased from Sigma-Aldrich (St. Louis, MO, USA). Conductive indium tin oxide (ITO)–coated microscope glass slides were purchased from Delta Technologies (Loveland, MN, USA) for the drug measurements. For the lipid measurements, SuperFrost Plus microscopic glass slides were purchased from VWR International BV (Radnor, PA, USA). All MALDI matrices, 2,5-dihydroxybenzoic acid ≥ 98% (DHB), 2,4,6-trihydroxyacetophenone hydrate ≥ 98% (THAP), α-cyano-4-hydroxycinnamic acid ≥ 98% (α-CHCA), 2′,6′-dihydrixyacetophenone (DHA), 1′,5′-diaminonaphthalene (DAN), norharmane, *N*-(1-naphthyl)ethylenediamine dihydrochloride (NEDC), and sinapic acid ≥ 98% (SA) were purchased from Sigma-Aldrich.

Different supportive tapes were purchased: double-sided conductive copper tape and double-sided carbon tape, double-sided Tesa® tape; double-sided Scotch® tape all manufactured by 3M (MN, USA).

### Sample collection

Male Wistar rats were obtained from the animal facility of Gasthuisberg (KU Leuven, Belgium) to facilitate targeted drug experiments by spiking blank bones with a methadone:EDDP (1:1) aqueous mixture. The animals served solely for breeding purposes and were not involved in any prior experiments. Samples of dosed rats were obtained from an experiment concerning chronic dosing of rats with methadone [20]. All applicable international, national, and institutional guidelines for the care and use of the animals were followed. All experiments were in accordance with ethical standards as approved by the Ethical Committee for Animal Experimentation of the University of Leuven (P 113/2011). The animals were euthanized using CO_2_. Bones were removed by dissection and stored at − 20 °C until future use. Human postmortem samples were obtained at autopsy of legal cases at UZ Leuven (Belgium). Approval for this study was received from the Medical Ethics Committee of the faculty of Medicine of the University Hospital of Leuven, Belgium. Cases were selected after a positive screening result for methadone. The clavicle was chosen as the specimen of choice due to the high accessibility during autopsy. After removal of the breastplate, a ring of 1-cm width was serrated 1 cm from the center of the distal clavicle head. The bones were cleaned by scraping the soft tissue off with a scalpel. Bone marrow was not removed prior to cutting. Samples were stored at − 20 °C.

For the untargeted lipid experiments, the control (not fractured) hind legs of a mouse study were used. These mice received pulsed electromagnetic field (PEMF) therapy for 0, 1, 4, or 8 h per day for 14 days. The animals were euthanized by cardiac puncture. The samples were stored on ice and transported to the freezer. Approval for this study was received from the Animal Experiments Committee of Maastricht University (2014-030). Before embedding and sectioning, the fur was removed, while the surrounding soft tissue and bone marrow remained. The samples were stored at − 80 °C.

### Tissue sectioning

Based on previous experiments [10], different embedding media were tested for thin rat bone sections: (A) an aqueous mixture of 20% gelatin, 5% CMC (w/v); (B) an aqueous mixture of 20% gelatin, 7.5% CMC (w/v); and (C) an aqueous mixture of 20% gelatin, 10% CMC (w/v). Also, different adhesive tapes were evaluated for supporting the tissue sections. Four different tapes were evaluated: double-sided conductive copper tape, double-sided carbon tape, double-sided Tesa® tape, double-sided Scotch® tape. For every tape separately, the optimal section thickness and temperature were determined. The sample was mounted on the specimen disc using 10% tragacanth (w/v). Tissue sectioning was performed on CryoStar NX50 (Thermo Scientific) or Leica CM1860 UV (Wetzlar, Germany) using a Shandon™ Tungsten Carbide D-Profile (Thermo Scientific). The tape was attached using the second adhesive side to a conductive indium tin oxide (ITO)–coated microscope glass slides from Delta Technologies (Loveland, MN, USA) for the drug experiment and on SuperFrost Plus microscopic glass slides (VWR International BV, Radnor, PA, USA) for the lipid experiments. For the lipid experiments, the samples were stored at − 80 °C until future use. The sample was vacuum-dried in a desiccator overnight at room temperature for the drug experiments and 30 min in the desiccator at room temperature for the lipid experiments before matrix application. For the application of the drug standards, it was necessary that the sections were completely dry to prevent water-based drops from spreading due to capillary forces while a short drying time for lipids is preferred due to the natural degradation of lipids at room temperature. When the sections are completely dried, drug standards with different concentrations of an aqueous methadone:EDDP mixture were applied to bone sections using manually spotting of 0.5-μL drops. The spiked sections were vacuum-dried for 15 min in a desiccator before matrix application.

### MALDI matrix selection with dried droplet

For the drug experiments, four matrices were evaluated and prepared as follows: 7 mg of DHB and 10 mg of NaCl dissolved in 1 mL of H_2_O/ACN (70:30 v/v); 10 mg of THAP and 10 mg of NaCl dissolved in 1 mL of MeOH; and 5 mg of SA and 1 mg of NaCl dissolved in 1 mL of H_2_O/ACN (40:60 v/v) and 5 mg of α-CHCA in 1 mL of H_2_O/ACN (70:30 v/v). Matrix solutions were deposited 1:1 (v/v; 0.5 μL each) together with a methadone:EDDP mixture on a stainless steel target and dried at room temperature. All experiments were performed in duplo.

### MALDI matrix sublimation

The different matrices were either sublimated on a home-built sublimation unit at TU Wien using experimental parameters as described previously [21] for preliminary experiments or on the HTX Sublimator (HTX Technologies, Chapel Hill, NC, USA). For the analysis of methadone and its metabolite, DHB was sublimated. For the matrix selection for the lipid experiments, CHCA, DAN, DHA, DHB, and norharmane were compared in positive ion mode, while DAN, NEDC, and norharmane were compared in negative ion mode. The sublimation details per matrix can be found in Table 1.

**Table 1:** Matrix sublimation details for the HTX sublimator unit. Per matrix, the amounts of matrix, solvent, sublimation temperature, and sublimation time are provided

Matrix	Amount (mg)	Solvent (± 5 mL)	Sublimation temperature (°C)	Sublimation time (s)
CHCA	55 ± 1	ACN:H_2_O (70:30)	180	300
DAN	30 ± 1	Methanol	120	80
DHA	50 ± 1	Isopropanol	160	300
DHB	50 ± 1 (lipid) 70 ± 1 (drug)	Acetone	180	280
NEDC	40 ± 1	Methanol	160	150
Norharmane	50 ± 1	Methanol	140	200

### MALDI-MSI instrumentation

A Waters SYNAPT G2 system was used for preliminary experiments before switching to the more sensitive SYNAPT G2-Si system equipped with a prototype uMALDI source, both provided with a Nd:YAG laser (Waters Corporation, Manchester, UK) (for more information about the uMALDI source, see Barré et al. [22]). The data acquiring was performed using MassLynx version 4.1 and HDImaging version 1.5 software (Waters Corporation). The measurements were performed in sensitivity mode with a scan rate of 1.0 s per scan. Different mass ranges were acquired: *m*/*z* 50–500 in positive ion mode for drug experiments, *m*/*z* 100–1200 in positive ion mode for lipid experiments, and *m*/*z* 100–2000 in negative ion mode for lipid experiments. The instrument was calibrated with red phosphorus for both positive and negative ion modes before each measurement. The laser fluence was dependent on the matrix used as well as the application type. It varied between 150 and 350 arbitrary units set in the control software, and no absolute fluence measurement was performed. The spatial resolution was 150 μm × 150 μm for the drug experiments and 100 μm × 100 μm for the lipid experiments.

### Data analysis

For the data analysis of the measurements, HDImaging (version 1.4, Waters Corporation), MassLynx (version 4.1, Waters Corporation), LipostarMSI (version 1.1.0b17, Molecular Horizon), and mMass (Open Source Mass Spectrometry Tool, version 5.5.0) were used. All data analysis is performed on total ion current (TIC)–normalized data and the shown distribution images are TIC-normalized.

### Lipid identifications

The selected *m*/*z* values (shown in the overlay images in “Results”) were chosen from the merged dataset created in LipostarMSI (version 1.1.0b17, Molecular Horizon). In this dataset, all measurements of one ion mode were merged and the *m*/*z* values were averaged over the different measurements. For each merged *m*/*z* value as displayed in the figure, the corresponding *m*/*z* value from the average mass spectrum for the specific matrix was obtained, as this value correlates to the shown distribution. These average mass spectra were recalibrated to the high-intensity matrix peaks and some general lipids using the manual mass warping option during data import in LipostarMSI. For tentative identifications of selected *m*/*z* values (from the average mass spectrum), the experimental *m*/*z* values were searched in the LIPID MAPS® Structure Database (LMSD) and ALEX^123^ lipid calculator with a maximum ppm error of 15 ppm, including all lipid classes and all single charged adducts [23, 24].

## Results

This study consists of three parts: (1) the optimization of a sample preparation method of undecalcified bone tissue for MSI, focused on the embedding material and sectioning method; (2) a targeted approach measuring drug concentrations in rat and human bone tissue, and more specifically on the detection of methadone and its metabolites to determine the limit of detection (LOD) and detection of these molecules in dosed bones; and (3) an untargeted approach to select the most suitable matrix for measuring lipids in mouse bone tissue.

### Method optimization for tissue sectioning

**Fig. 1:**
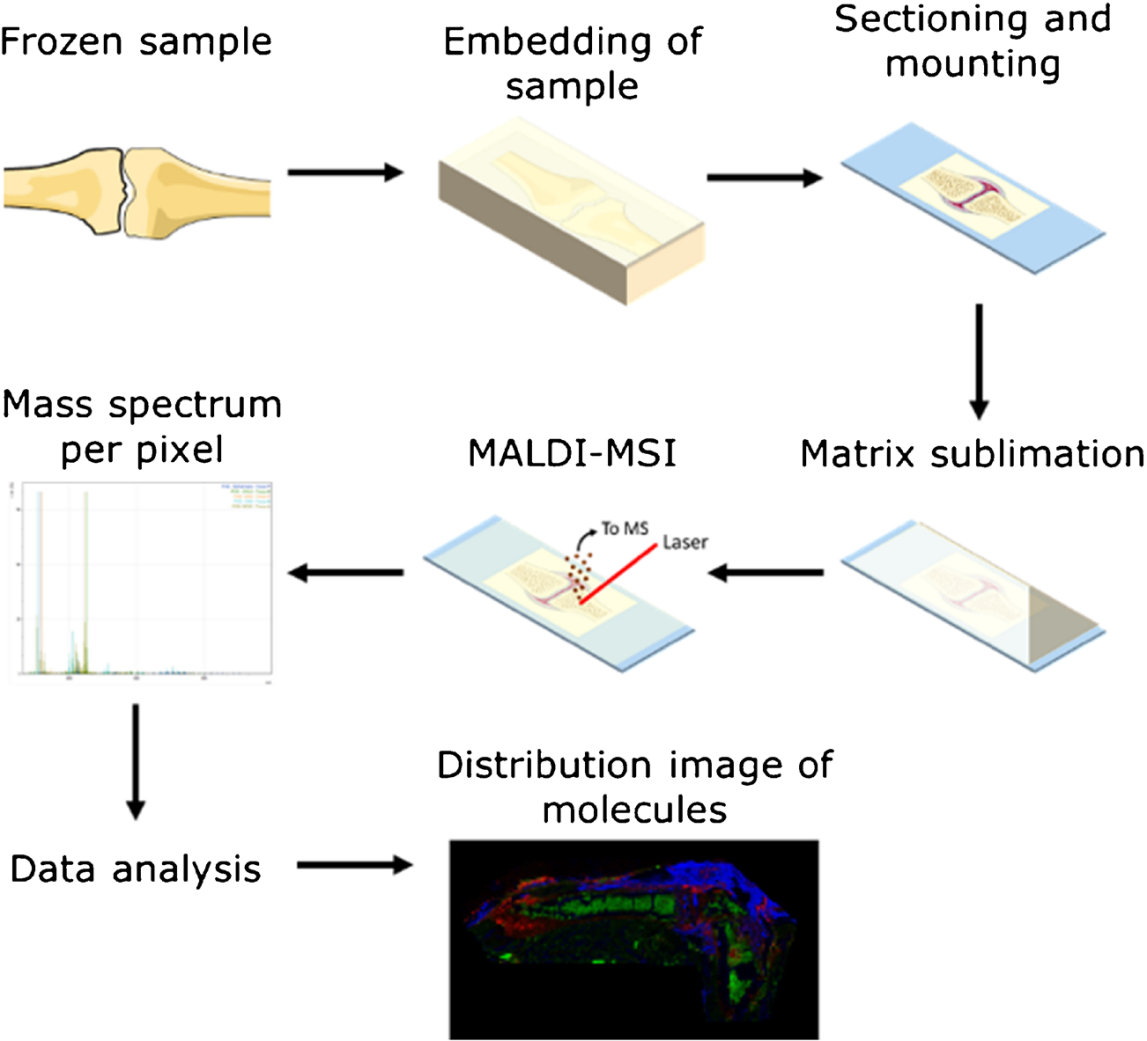
Analytical workflow for the analysis of bone tissue with MALDI-MSI. Samples were collected and stored in the freezer until further use. Samples were embedded in 20% gelatin with 7.5% CMC (w/v), sectioned at 12 μm with the support of double-sided tapes, and mounted with the other side of the tape on a glass slide such that the tissue was available for analysis. The matrix was applied on the sample via sublimation. After matrix sublimation, the MALDI-MSI measurement was performed, during which a mass spectrum per pixel is obtained for the region of interest. After data processing and analysis of these spectra, for example, intensity and signal-over-noise (S/N) values could be obtained and distribution images of selected *m*/*z* values could be created that can provide information about the location of these molecules

Cutting undecalcified bone tissue without embedding resulted in shattered fragments. Therefore, embedding bone tissue proved necessary to maintain sample integrity during sectioning. In a previous study, gelatin and CMC has been used as embedding materials for chicken digits [10]. Chicken digits differ from rat and mouse bone, the latter being harder and more brittle. An optimal embedding material and sectioning protocol for undecalcified bone tissue of rats and mice focusing only on MALDI-MSI has not been determined yet. Three different embedding media were tested with different concentrations of CMC to maintain the integrity of the sample. Of these embedding media, 7.5% proved to provide the best sections, while 10% CMC showed to exceed the solubility threshold. Tissue sections were visually assessed by a light microscope to evaluate their integrity. Optimal section parameters were chosen based on a surface homogeneity. As the embedded samples still shattered during sectioning, double-sided tapes were used to increase the support. The section thickness and temperature were optimized for each of the four tapes. Optical images of a section obtained with different tapes are shown in ESM Fig. S1. The Tesa® tape gave the most homogenous sections. The final workflow for the sample preparation and MALDI-MSI measurements for undecalcified bone tissue is shown in Fig. 1.

### Targeted detection of methadone and EDDP

#### Matrix selection for drugs

The limit of detection (LOD) was determined based on a signal-to-noise (S/N) ratio of more than 3. In a common MALDI-MS experiment using the dried droplet method on a stainless steel target, the LOD was estimated to approximately 2.5 pg for methadone and 1 pg for EDDP using DHB as a matrix. Mass spectra are shown in ESM Fig. S2. Using SA as a matrix, the LODs were estimated to approximately 50 pg for methadone and 30 pg for EDDP. CHCA showed to have an overlapping mass with EDDP. THAP did not co-crystallize with methadone and gave no signal. The amount of DHB for detection of methadone and EDDP was optimized, resulting in 70 mg of DHB. This latter resulted in 3.3 mg DHB on the ITO slide.

#### Sensitivity test

**Fig. 2:**
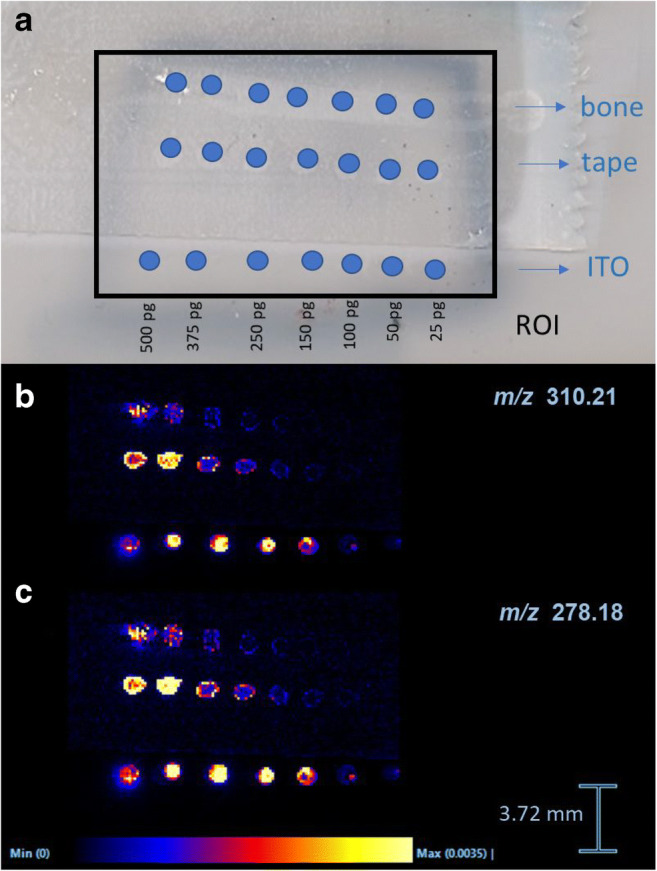
Calibration series for methadone and EDDP. **a** Longitudinal bone section spiked with methadone dilution series region of interest (ROI) in blue. (**b**) MALDI-MSI image of methadone (*m*/*z* = 310.21) and (**c**) EDDP (*m*/*z* = 278.18). The distribution images are total ion current (TIC)-normalized. Scale bar shows relative intensities

On the SYNAPT G2-Si, the copper tape as well as the normal tape gave sufficient signal intensities. Because the quality of the sections was better using the normal Tesa® tape, the latter was used for future experiments. Different calibration series were constructed to compare the influence of the different parameters (ITO slide, tape, and bone tissue). Methadone and EDDP were detectable as [M+H]^+^ ions in MS mode in all calibration series. A significant decrease in intensity could be observed from bone tissue compared with ITO glass slides exemplifying the significantly reduced desorption/ionization efficiency of analytes. The LOD of detection for methadone was estimated to approximately 5 pg, 25 pg, and 50 pg in a drop on the ITO slide, the tape with gelatin, and the bone tissue, respectively. For EDDP, LODs were estimated to approximately 25 pg on bone, 25 pg on tape with gelatin, and 5 pg on the ITO slide. On the ITO slide and the tape, methadone and EDDP generated characteristic ion fragment signatures in MS/MS mode. On the bone tissue, below 500 pg, no specific MS/MS spectra could be generated. The calibration series are shown in Fig. 2. The mass spectra of a 50-pg spot on bone tissue are shown in ESM Fig. S3.

#### Detection of methadone and metabolite in dosed rat bone

**Fig. 3:**
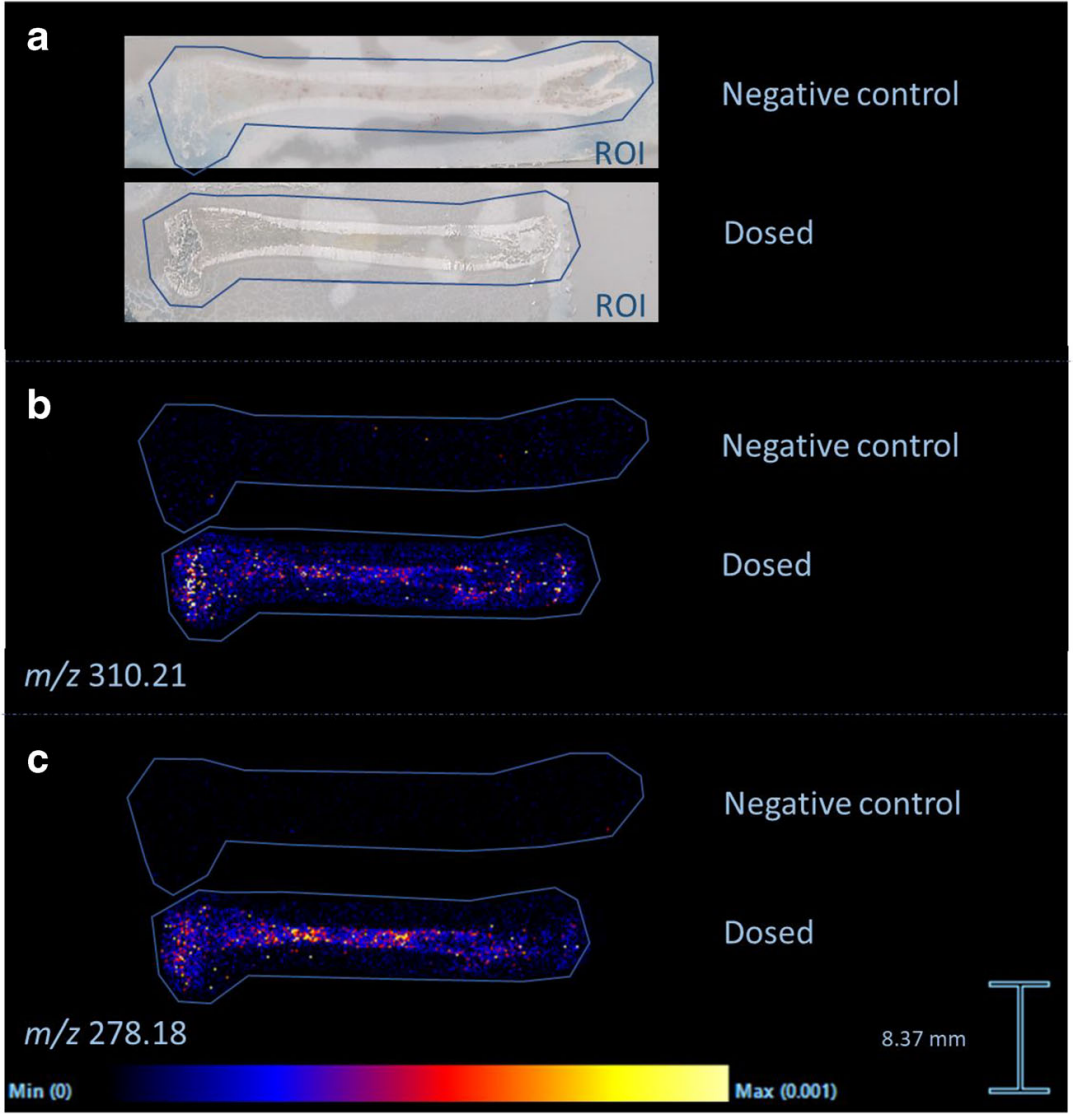
Distribution images of methadone and EDDP in a rat femur dosed with methadone. **a** Longitudinal section of rat femur with region of interest (ROI) in blue. **b** MALDI-MSI distribution image of methadone (*m*/*z* 310.21). **c** MALDI-MSI distribution image of EDDP (*m*/*z* 278.18). The distribution images are total ion current (TIC)-normalized. Scale bar shows relative intensities

A femoral bone of a rat dosed with methadone was sectioned longitudinal and imaged as described. The methadone concentration and EDDP concentration in the bone were determined respectively at 14 ng/g and 4 ng/g as described by Vandenbosch et al. [20]. The intensity of methadone in the bone was very low. The methadone intensity in the bone marrow could clearly be distinguished from the surrounding bone. The surrounding bone showed a slight intensity difference with the surrounding matrix, but this difference is not significant. For the metabolite EDDP, the same pattern was seen and the signals were more intense at some spots. The bone tissue could be distinguished from the surrounding matrix, but this difference is not significant. The distribution images of methadone and EDDP are shown in Fig. 3.

#### Detection of methadone and metabolite in postmortem forensic human bone

**Fig. 4:**
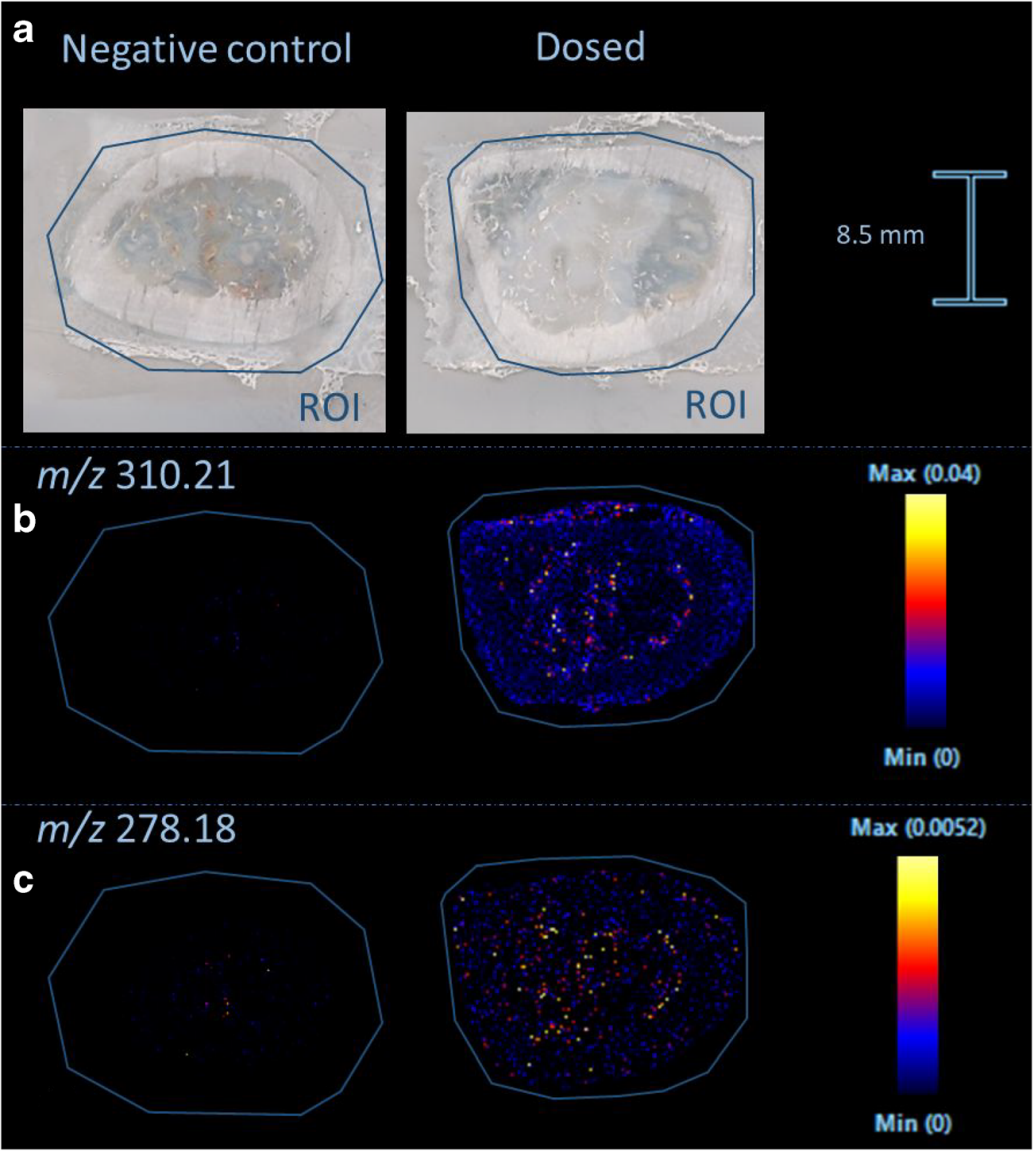
Distribution images of methadone and EDDP in a human clavicle from someone who overdosed on methadone. **a** Cross section of the clavicle with the region of interest (ROI) in blue. **b** MALDI-MSI distribution image of methadone (*m*/*z* 310.21). **c** MALDI-MSI distribution image of EDDP (*m*/*z* 278.18). The distribution images are total ion current (TIC)-normalized. Scale bar shows relative intensities

A clavicular bone of a 37-year-old male who died of an overdose of methadone was sectioned cross-sectional and imaged as described. Blood concentrations of methadone and EDDP were respectively 3727.4 ng/mL and 32.9 ng/mL as described in Vandenbosch et al. [25]. When looking at the distribution image of methadone, the bone marrow could be distinguished from the surrounding bone (see Fig. 4). In addition, the bone tissue could clearly be distinguished from the surrounding matrix. Similar observations were made for EDDP (see Fig. 4).

### Untargeted detection of lipids

In previous studies of MALDI-MSI on undecalcified bone tissue, CHCA, DHB, and dithranol have been reported as matrices for the detection of metabolites and lipids from bone tissue, but limited information is available on lipids and the efficiency of these matrices to desorb and ionize different lipid classes [7, 9, 10]. Studying the different lipid classes present in bone is of great interest, because of their regulatory role in bone health and repair [7, 19]. To test the different matrices for the detection of lipids in bone tissue, the following matrices were assessed: CHCA, DAN, DHA, DHB, and norharmane for positive ion mode; and DAN, NEDC, and norharmane in negative ion mode. Herein, we focus on positive ion mode. Results for negative ion mode are provided in the ESM in section Information S1 with the corresponding figures and tables.

**Fig. 5:**
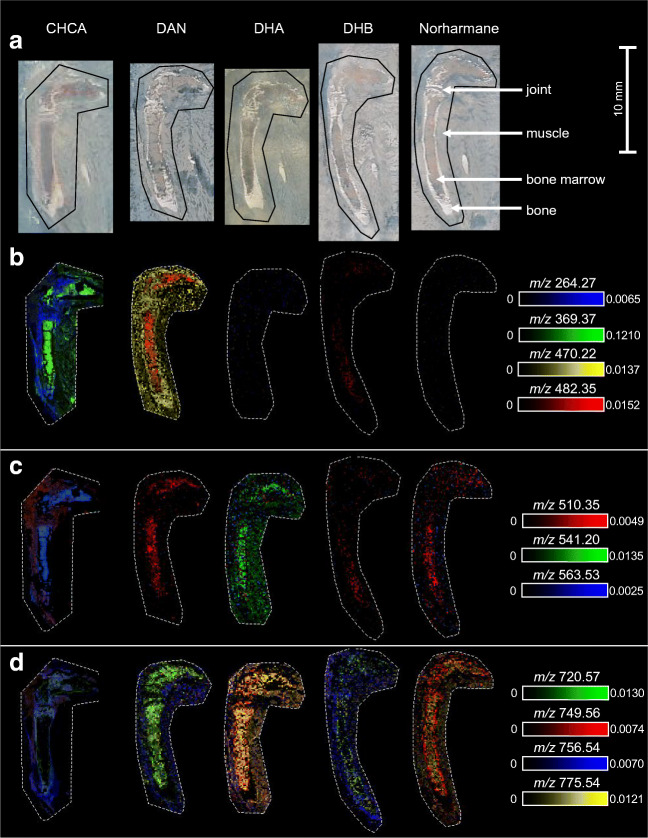
Overlay images of selected *m*/*z* values for CHCA, DHA, DAN, DHB, and norharmane in positive ion mode measured with MALDI-MSI of mouse hind legs for the untargeted detection of lipids. The *m*/*z* values were selected to show the similarities and differences between the matrices in terms of desorption and ionization efficiency. **a** Optical scan of the section with the measured area indicated with a black line. Overlay images of the distributions of selected *m*/*z* values in the range 200–500 (**b**), 500–600 (**c**), and 700–800 (**d**). The distribution images are total ion current (TIC)-normalized and the intensity scale shows the maximum relative intensity of the specific *m*/*z* value (for tentative identifications of the selected *m*/*z* values, see ESM Table S3)

In positive ion mode, different matrices allowed for the detection of different molecules from the bone and bone marrow, although some *m*/*z* values were represented in multiple matrices (see Fig. 5). For DAN, DHA, and norharmane, it was not possible to obtain specific *m*/*z* values from bone, although for norharmane some *m*/*z* values showed higher intensities in bone tissue compared with the bone marrow and surrounding tissue. For CHCA and DHB, it was possible to obtain *m*/*z* values specific for bone and bone marrow. In addition, CHCA and DHB had the highest number of *m*/*z* values specific for bone or bone marrow (see ESM Table S2). DAN and norharmane resulted in a high number of specific *m*/*z* values for bone marrow compared with DHA. For CHCA, the specific *m*/*z* values were roughly half divided between bone and bone marrow, while for DHB, the majority of the specific *m*/*z* values were obtained from bone (see ESM Table S2). DHB has relatively low signal intensities (TIC-normalized) and signal-to-noise (S/N) ratios (see ESM Table S2 and mass spectra in ESM Fig. S4) for bone as well as bone marrow compared with the base peak, while CHCA has low signal intensities and S/N ratios for bone in contrast to data collected from bone marrow. DAN provided the highest S/N ratios for signals derived from bone marrow, but in the positive ion mode, this matrix showed significant interference with the tissue-related peaks. DHB and norharmane show some interferences, and for CHCA and DHA, the interference is low (ESM Fig. S4).

## Discussion

### Method optimization for sectioning of undecalcified bone tissue

Here, we present a sample preparation protocol for undecalcified bone tissue developed and optimized for analysis with MALDI-MSI. Embedding in an aqueous solution of 20% gelatin (w/v) and 7.5% carboxymethyl cellulose (CMC, w/v) in combination with the use of double-sided tapes proved to be necessary to maintain sample integrity during sectioning. It is of utmost importance to avoid bubbles of any kind (air, water from melting crystals in tissue) in the medium during embedding of the tissue. Bubbles will result in holes in the embedding which cause less support during sectioning and thus low-quality sections, while at 60 °C, the embedding material is poured in a mold. The tissue sample is immersed immediately. As a result, the frozen sample will start melting. It is recommended to immediately start polymerization after sample embedding by freezing at − 20 °C to avoid tissue degeneration due to the heat. Only the tungsten carbide knife was found to be robust enough to cut bone tissue without permanent deformation of the blade. Different amounts of CMC were tested. The amount of CMC was increased compared with that in previous studies to ensure a smooth transition from embedding to bone tissue while sectioning. Increased amounts of CMC gave more homogenous sections with less relief compared with lower CMC concentrations. Above 7.5% (w/v), the solution was saturated and CMC could not dissolve anymore. Still the sections were not completely homogenous. An embedding medium with characteristics closer to those of bone tissue would have been even better, but this medium still has to be MSI-compatible, making conventionally used resins unsuitable. For example, other research groups use tragacanth or SCEM-L1 embedding medium for sectioning bone tissue [7, 26]. Unfortunately, tragacanth is hygroscopic, making it not fit for use after storage in a freezer. On the contrary, the gelatin/CMC-embedded samples could be easily reused for at least 5 times after storage. Samples were stored in aluminum foil and frozen at − 20 °C or − 80 °C. Although the gelatin/CMC mixture is MS-compatible, it has the disadvantage of being used at higher temperature than the frozen tissue during embedding, which can cause heat stress and/or affect the molecular composition of the bone [11].

Four different double-sided tapes were evaluated for suitability, specifically two non-conductive normal tapes and two conductive tapes. When choosing a tape as a support during sectioning, different parameters should be taken into consideration. The first one is the capability of the tape to stick to the frozen gelatin/CMC block. While the temperature affects the adherence of the tape, it is important to have a good adherence to the frozen sample to create a homogenous sample. As a result, the adherence will determine the optimal temperature for sectioning as well as the thickness of the sections. A tape with a good adherence makes it possible to generate thinner sections. In addition, the thickness of the sections should be considered carefully. On one side, a thin section will give less insulation and hence a higher overall signal intensity. On the other side, thinner sections also mean that less analyte will be available for extraction into the MALDI matrix and subsequent desorption [1]. Thicker sections will take more time to dry in the desiccator. It is noteworthy that some mass spectrometers, such as linear ToF, conductive samples/tape, and therefore the tape selected here, would be unsuitable. The double-sided Tesa® tape resulted in the most homogenous sections at a thickness of 12 μm.

### Targeted detection of methadone and EDDP

The targeted detection of methadone and its metabolite EDDP requires the right matrix which crystallizes with the chosen analyte but does not provide too many background peaks overlapping with the peaks of interest. This is important since most matrices show molecular ion signals, multiple adduct ions, as well as aggregates in the lower mass range (*m*/*z* < 1000). A method for detection and visualization of methadone and its metabolites was developed and optimized. Matrix selection was performed using the dried droplet technique. Four common matrices were tested, of which DHB gave the best results. DHB showed the lowest LOD of 2.5 pg for methadone and 1 pg for EDDP using DHB as a matrix. Additionally, no interfering peaks with the small molecules from methadone and EDDP were found. SA showed to ionize the analyte of interest, but increased LODs. CHCA also showed to ionize the analyte of interest but an interfering peak was close to the mass of the metabolite EDDP. THAP did not work for this application. The sensitivity of MALDI-MSI technology was tested by investigating the LODs. Significant ion suppression was noticed when spiking standards on bone tissue compared with ITO slides. In this study, a deliberate choice was made for a sample preparation protocol without any pretreatment of the bone tissue to reduce interfering molecules or other contaminants. This is required to maintain the molecular distribution and to retain the presence of minerals and lipids in the bone tissue. As lipids desorb/ionize relatively easily, they are the dominant fraction of ions in every MALDI-MSI spectrum. Thereby, lipids suppress molecular ion fractions of lower abundance or molecular ion fractions that desorb/ionize with more difficulty [27]. The intensities of methadone and EDDP decrease on the tape and even further on the bone tissue when compared to the ITO glass slide (Fig. 2). The obtained findings indicate that bone tissue and the supporting tape have a significant negative effect on the grade of desorption/ionization. These negative effects can partly be accounted for by the high content of minerals, e.g., hydroxyapatite, and lipids present in the bone. When comparing the intensities between the spots on tape and those on the ITO slide, also a diminishing effect on the desorption/ionization rate is seen solely due to the tape.

Another factor of impact could be height differences in the sample surface. The samples consisted of heterogeneous tissues such as bone tissue, bone marrow, and even surrounding tissue. Due to drying, the thickness of the section can change in nanometers. Bone does not shrink when dried, but bone marrow does. As a result, minor peak shifts can occur. This problem was countered by the usage of an orthogonal ToF system, in which the source is decoupled from the mass analysis. This causes the mass analysis to be independent of the height of the sample, making it better suited for the analysis of non-homogenous samples, such as bone [28]. Nevertheless, ionization efficiency may be affected as the laser is not optimally focused on each part of the tissue if height differences occur [29].

In the MALDI-MSI experiments, methadone (*m*/*z* 310.21) and EDDP (*m*/*z* 278.18) were distinguishable from background signals in the bone marrow of the rat and human bone (see Figs. 3 and 4). Confirmatory MS/MS experiments were not performed due to the low intensities of our analytes, but high mass accuracy of the oToF system strongly corroborates our so far tentative assignment. Internal calibration of the instrument was performed daily in the mass range 50–650 Da (< 1 ppm) using red phosphorus. Afterwards, the mass accuracy was checked with quality control samples in the form of calibration series spiked on blank bone sections. Aside to that, the untreated and dosed bones were analyzed in the same experimental run to ensure a consistent mass accuracy.

When looking at the cross section of human bone tissue, it still looked slightly shattered. This is in part caused by the histological structure of bone tissue. Compact bone mostly consists of osteons or Haversian systems, which are cylindrical structures. They are aligned parallel to the long axis of the bone. Therefore, sections made in parallel with the osteons give the best results, while sectioning perpendicular to the osteons causes fragmentation, as seen in the human clavicle. Another important factor is that the sample sectioning method is optimized using rat bone. Rat bone is less hard than human bone which causes more fractions in the human sections.

### Untargeted detection of lipids

As only a few studies have been performed on undecalcified bone using MALDI-MSI, it is important to compare different matrices in their ability to desorb and ionize a broad range of lipids from bone and bone marrow. Different lipid classes are of interest in (pre)clinical research, because of their important regulating role in maintaining bone health and fracture healing [7, 19]. This broad range of lipids can contribute to improve the understanding of the lipid pathways in bone healing and impaired bone healing. Here, different matrices were compared based on the number of specific *m*/*z* values from bone as well as bone marrow, the intensity of the specific *m*/*z* values in comparison with the matrix peaks, the amount of background signal from the matrix, and the interference of the matrix peaks with signal from the tissue.

In positive ion mode, five matrices for measurement of lipids were compared, namely CHCA, DAN, DHA, DHB, and norharmane. Of these matrices, DAN was the only matrix that showed an unwanted reaction with components of the double-sided tape over time after the measurement was performed. The tape became untransparent, which can cause complications with staining of the same section after the MALDI-MSI measurement. Besides, DAN showed the highest interference of the matrix and background peaks with the tissue-related peaks of all the matrices (ESM Table S2). For all matrices in positive ion mode, it was possible to obtain ion distribution images up to *m*/*z* 800 (see Fig. 5). The bone- and bone marrow–specific *m*/*z* values could be found over the complete measured mass range, although these intensities were lower for *m*/*z* values above 800. This broad range of values indicated that different lipid classes could be obtained with these matrices, which have been shown to be present in bone and bone marrow and contribute to bone health [19]. With DAN, DHA, and norharmane, no specific *m*/z values could be obtained from only bone tissue in the 2000 most abundant peaks, which makes these matrixes less suitable for the (pre)clinical research related to fracture healing. CHCA and/or DHB showed to be the best matrices for the analysis of lipids in bone and bone marrow, as these matrices resulted in the highest number of *m*/*z* values specific for bone as well as bone marrow with low interference of the matrix and background peaks with the tissue-specific peaks. Nevertheless, the intensities and S/N values of the specific *m*/*z* values for bone and bone marrow are relatively low.

In Fig. 5, it can be seen that most of the selected *m*/*z* values are not present in all the different matrices. Most of the detected *m*/*z* values specific for bone or bone marrow were found in only one of the compared matrices or showed different distributions between matrices, for example, surrounding muscle tissue versus bone marrow. This can indicate that the detected molecules at the same *m*/*z* value might not be the same between matrices, emphasizing the fact that the desorption and ionization mechanism of each matrix is different, resulting in the ionization of different lipid classes [30]. Therefore, matrix selection depends on the lipid class of interest. For positive ion mode, CHCA and DHB showed to be able to absorb and ionize a broad range of lipid classes from bone and bone marrow. These matrices could be used in future preclinical studies about different skeletal diseases.

For most of the matrices, the intensities and S/N values for the detected molecules were relatively low, especially compared with the high intensities of matrix peaks. It is expected to obtain an improvement in these values by applying the matrix by spraying it with an automated sprayer. Spraying the matrix is a wetter matrix application method allowing for a better extraction of analytes from the tissue [31]. In addition, trifluoroacetic acid (TFA) can be added when spraying CHCA or DHB to further enhance the extraction of molecules from the tissue [2, 27]. An advantage of matrix sublimation in comparison with spraying of a matrix is the lower diffusion of analyte and, therefore, less delocalization [27]. In addition, sublimation results in smaller crystals than when a matrix is sprayed, which can be an advantage for measurements at lower spatial resolution [27]. In general, the application of a matrix by sublimation is faster than spraying and should provide good detection of different lipid classes, which is why for this study sublimation was selected. Nevertheless, whether or not spraying of the matrix will improve detection and intensity of different lipid classes for bone tissue should be studied.

## Conclusion

This study describes the optimization of the sample preparation protocol for MALDI-MSI of undecalcified bone tissue in close contact with soft tissue like bone marrow or muscle tissue. Embedding of the sample in combination with a supportive tape to transfer the section to the sample holder is necessary to obtain high-quality bone sections. As height differences may occur between bone and bone marrow, mass spectrometers that are not affected by these differences are more appropriate for analysis of bone tissue. Experiments can be performed on mass spectrometers where the ionization is decoupled from the mass analysis. This optimized bone tissue preparation protocol for MALDI-MSI was applied in a targeted and untargeted approach. The sample preparation protocol was shown to be applicable for the targeted detection of methadone and its metabolites as well as the untargeted detection of lipids from bone tissue. MALDI-MSI has been used to estimate the limits of detection and the distribution of the methadone and its metabolites in rat and human bone tissue. In addition, CHCA and/or DHB showed to be the best matrix for lipid detection in positive ion mode. The optimized sample preparation protocol and these targeted and untargeted approaches clear the path for future forensic and (pre)clinical investigations.

## Electronic supplementary material

ESM 1 (PDF 1406 kb)
