# Supplementary material for: Sample preparation of bone tissue for MALDI-MSI for forensic and (pre)clinical applications
Source: Anal Bioanal Chem. 2020 Sep 15;413(10):2683–94. doi: 10.1007/s00216-020-02920-1 (PMC8007508; doi:10.1007/s00216-020-02920-1)
Supplement: Supplementary file 1 — (PDF 1406 kb) [file 216_2020_2920_MOESM1_ESM.pdf]

Analytical and Bioanalytical Chemistry

Electronic Supplementary Material

Sample preparation of bone tissue for MALDI-MSI for forensic and (pre)clinical applications

Michiel Vandenbosch, Sylvia P. Nauta, Anastasiya Svirkova, Martijn Poeze,
Ron M.A. Heeren, Tiffany Porta Siegel, Eva Cuypers, Martina Marchetti-Deschmann

Index

Table S1: Overview of MALDI-MSI and similar techniques studies performed on bone tissue.

Table S2: Comparison of CHCA, DAN, DHA, DHB, and norharmane in positive ion mode using mice bone for the untargeted application.

Table S3: Tentative identifications of selected *m/z* values in positive ion mode for the untargeted application.

Figure S1: Optical images of rat bones of each of the four tapes used during sectioning for sample preparation optimization.

Figure S2: Mass spectrum of a droplet with a drug mixture of 1pg EDDP and 2.5 pg methadone

Figure S3: Mass spectrum of a spot with 50 pg methadone and 50 pg EDDP on bone tissue

Figure S4: Profile mass spectra of the measured area for the different matrices in positive ion mode using mice bone for the untargeted application.

Information S1: Untargeted application for lipids in negative ion mode using mice hind legs.

Table S4: Comparison of DAN, NEDC, and norharmane in negative ion mode using mice bone for the untargeted application.

Figure S5: Overlay images of selected *m/z* values for DAN, NEDC, and norharmane in negative ion mode of mice bone for the untargeted application.

Figure S6: Profile mass spectra of the measured area for the different matrices in negative ion mode using mice bone for the untargeted application.

Table S5: Tentative identifications of selected *m/z* values in negative ion mode for the untargeted application.

Table S1 Overview of MALDI-MSI and similar techniques studies performed on bone tissue. Information is provided about the sample, sample preparation (pretreatment, embedding material, sectioning specifications, and washing), MSI specification, and results

Abbreviations: APS-MALDI-MSI = atmospheric pressure scanning microprobe matrix-assisted laser desorption/ionization mass spectrometry imaging, CMC = carboxymethyl cellulose, IR-MALDESI-MSI = infrared matrix assisted laser desorption electrospray ionization mass spectrometry imaging, MALDI-MSI = matrix-assisted laser desorption/ionization mass spectrometry imaging, MSI = mass spectrometry imaging, PFA = paraformaldehyde, SIMS = secondary ion mass spectrometry, TCA = trichloroacetic acid, µXRF = micro X-ray fluorescence

Author (year)	Sample	Pretreatment	Embedding material	Sectioning specifications	Washing	MSI specifications	Results
**Seeley, *et al.* (2014)**[1]	Mice with tumor-induced bone disease	-	Ice	Whole body sections<br>Thickness: 15 µm<br>Sections collected using Instrumedics, Inc. Macro Tape Transfer system (Leica), whereby a polymer is used to transfer section from tape to slide	70%, 90%, and 95% ethanol for 30 s each	MALDI-MSI, positive ion mode, *m/z* range 2,000-40,000	Mass spectra could be acquired from bone marrow, but not from bone.
**Fujino, *et al.* (2016)**[2]	Mice femurs and tibia	Decalcification using formic acid, $EDTA\text{-}NH_4$, or TCA.	2% CMC	Kawamoto method<br>Thickness: 10 µm<br>Tape: Electrical conducting double-	70% and 100% ethanol	MALDI-MSI, positive and negative ion mode, *m/z*	Each decalcification and/or fixation method has its own characteristic peaks. No significant difference in

		Fixation using 4% PFA, Carnoy fluid, or TCA.		sided tape		range of 100-1000	number of peaks per method. Using cryotape reduces the number of peaks in the mass spectra.
**Scheape, *et al.* (2018)**[3]	Human femoral heads	-	First, big blocks in 4% PHA. Second, smaller pieces in SCEM-L1 embedding medium from SECTION-LAB Co.	Kawamoto method<br>Thickness: 4 µm<br>Knife: SL-30 tungsten carbide blade<br>Tape: cryofilm of type 2C(9) from SECTION-LAB Co.	-	APS-MALDI-MSI, positive ion mode, *m/z* range 250-1000<br>SIMS, positive and negative ion mode,	Different distributions of major lipid classes could be shown, including glycerolipids, glycerolphospholipids, and sphingolipids. In addition, triacylglycerols could be found in bone marrow.
**Svirkova, *et al.* (2018)**[4]	Chicken digits	-	20% gelatin with 5% CMC	Ullberg method<br>Thickness: 12 µm<br>Knife: non-disposable tungsten carbide knife<br>Tape: double-sided tape and polyimide one sided tape made with DuPont^{TM} Kapon®	-	MALDI-MSI, positive ion mode, *m/z* range 300-1000<br>µXRF	Lipid distributions were shown in surrounding tissues using MALDI-MSI and elements in the bone using µXRF.

**Khodjaniyazova, *et al*. (2019)**[5]	Mice humeri	-	Half of the bone in plaster of Paris, in addition, other embedding materials were tested	The cutting surface at half the bone was imaged. The bone was trimmed using a Surgipath high-profile disposable blade at 20 μm).	-	IR-MALDESI-MSI, positive ion mode, *m/z* range 250-1000	Lipid distribution images were shown from bone tissue, including a ceramide, phospholipid, and cholesterol.

Table S2 Comparison of CHCA, DAN, DHA, DHB, and norharmane in positive ion mode using mice hind legs for the untargeted application. The number of specific *m/z* values is given for bone as well as bone marrow, which are the number of specific *m/z* values from the 2000 most intense peaks as selected by HDImaging (version 1.4, Waters Corporation). From these specific *m/z* values, values potentially related to the matrix and background were removed by comparing them to a measurement of the background. The average signal-to-noise (S/N) is calculated for the specific *m/z* values and the ratio to the background is calculated in comparison to the highest background peak, based on the S/N-values obtained after peak picking using mMass (Open Source Mass Spectrometry Tool, version 5.5.0). In addition, the interference of the matrix with signals from the tissue is given based on the average mass spectra looking at the amount of background signal and the overlap of background peaks with peaks specific for tissue

**Matrix**	**Specific *m/z* values**			**Signal-to-noise (S/N) ratio**				**Interference of matrix with tissue signal**
	Bone	Bone marrow	Total	Average bone	Ratio to background (%)	Average bone marrow	Ratio to background (%)	
**CHCA**	50	57	107	257.62	0.04	895.20	0.14	Low
**DAN**	0	35	35	-	-	966.37	0.29	High
**DHA**	0	5	5	-	-	388.13	0.17	Low
**DHB**	96	7	103	223.51	0.05	81.51	0.02	Middle
**Norharmane**	0	23	23	-	-	138.22	0.09	Middle

Table S3 Tentative identifications of selected *m/z* values in positive ion mode. Per *m/z* value from the merged dataset, the experimental *m/z* value from the average mass spectrum of the corresponding matrix, the *m/z* value for a random pixel (from the corresponding matrix), the matched molecular formula, the theoretical *m/z* value, the Δm ($m/z_{exp} - m/z_{theo}$), the ppm error ($\frac{m/z_{exp}-m/z_{theo}}{m/z_{theo}} \times 10^6$), the potential matching lipid(s), and the detected ion are provided

Abbreviations lipid classes: CerP = ceramide phosphate; CerPE = glycerophosphoethanolamine ceramide; DAG = di(acyl/alkyl)glycerol; EPC = ethanolaminephosphoceramide; FA= fatty acids; HexCer = hexosyl ceramide; LIPC = lyso-inositolphosphoceramide; LPI = lyso-glycerophosphoinositols; MGDG = monogalactosylmonoacylglycerol; MGMG = monogalactosylmonoacylglycerol; PA = glycerophosphate; PAA = glycerophospholipid class; PC = glycerophosphocholine; PE = glycerophosphoethanolamine; PG = glycerophosphoglycerols; PI = glycerophosphoinositols; PS = glycerophosphoserine; SM = sphingomyelin; SPB = sphingoid base; SQMG = sulfoquinovosyl monoacylglycerol; ST = sterols; TAG = tri(acyl/alkyl)glycerol

**Merged dataset *m/z* value***	**Experimental *m/z* value average spectrum (matrix)**	***m/z* value random pixel (matrix)**	**Molecular formula**	**Theoretical *m/z***	**Δm**	**Ppm error**	**Possible lipid**	**Ion**
**264.2711**	264.2712 (CHCA)	264.2808 (CHCA)	$C_{18}H_{34}N$	264.2686	0.0026	9.84	SPB 18:2;O	$[M+H-H_2O]^+$
**369.3721**	369.3721 (CHCA)	369.3763 (CHCA)	$C_{24}H_{49}O_2$	369.3727	-0.0006	-1.62	FA 24:0	$[M+H]^+$
**470.2184**	470.2084 (DAN)	470.2063 (DAN)	$C_{19}H_{37}NO_{10}P$	470.2150	-0.0066	-14.04	LIPC 13:2;O2	$[M+H]^+$
**482.3470**	482.3409 (DAN)	482.3486 (DAN)	$C_{27}H_{48}NO_6$	482.3476	-0.0067	-13.89	ST 27:2;O6	$[M+NH_4]^+$
**510.3497**	510.3694 (DAN)	510.3800 (DAN)	$C_{25}H_{52}NO_9$	510.3637	0.0057	11.17	MGMG 16:0	$[M+NH_4]^+$
**541.2046**	541.2724 (DHA)	541.2345 (DHA)	$C_{24}H_{45}O_{11}S$	541.2677	0.0047	8.68	SQMG 15:1	$[M+H]_+$
			$C_{24}H_{46}O_{11}P$	541.2772	-0.0048	-8.87	LPI 15:0	$[M+H-H_2O]^+$
			$C_{27}H_{41}O_{11}$	541.2643	0.0081	14.96	ST 21:2;O5;GlcA	$[M+H]^+$
**563.5326**	563.4944 (CHCA)	563.5432 (CHCA)	$C_{34}H_{68}O_4Na$	563.5010	-0.0066	-11.71	DAG O-31:0	$[M+Na]^+$
**720.5671**	720.5701 (DAN)	720.5379	$C_{41}H_{80}NO_5PNa$	720.5667	0.0034	4.72	CerP 41:2;O	$[M+Na]^+$
			$C_{41}H_{79}NO_7Na$	720.5749	-0.0048	-6.66	HexCer 35:1;O	$[M+Na]^+$
			$C_{38}H_{79}N_3O_7P$	720.5650	0.0051	7.08	CerPE 36:2;O3<br>EPC 36:2;O3<br>SM 33:2;O3	$[M+NH_4]^+$
			$C_{43}H_{78}NO_7$	720.5772	-0.0071	-9.85	TAG 40:4;O	$[M+NH_4]^+$
			$C_{39}H_{78}NO_{10}$	720.5620	0.0081	11.24	HexCer 33:0;O4	$[M+H]^+$
			$C_{39}H_{78}NO_{10}$	720.5620	0.0081	11.24	MGDG 30:0	$[M+NH_4]^+$
**749.5561**	749.5592 (DHA)	749.4490 (DHA)	$C_{41}H_{79}N_2O_6PNa$	749.5568	0.0024	3.20	SM 36:3;O2	$[M+Na]^+$

			$C_{41}H_{82}O_9P$	749.5691	-0.0099	-13.21	PG O-35:1	$[M+H]^+$
			$C_{46}H_{78}O_6Na$	749.5691	-0.0099	-13.21	TAG 43:5	$[M+Na]^+$
			$C_{46}H_{78}O_5K$	749.5481	0.0111	14.81	DAG 43:6	$[M+K]^+$
			$C_{44}H_{78}O_7P$	749.5480	0.0112	14.94	PA 41:4	$[M+H-H_2O]^+$
			$C_{44}H_{78}O_7P$	749.5480	0.0112	14.94	PA O-41:6	$[M+H]^+$
**756.5372**	756.5274 (CHCA)	756.5330 (CHCA)	$C_{40}H_{80}NO_7PK$	756.5304	-0.0030	-3.97	PC O-32:1<br>PE O-35:1	$[M+K]^+$
			$C_{39}H_{75}NO_{11}Na$	756.5232	0.0042	5.55	HexCer 33:1;O5	$[M+Na]^+$
			$C_{45}H_{75}NO_6P$	756.5326	-0.0052	-6.87	PE O-40:8	$[M+H-H_2O]^+$
			$C_{48}H_{70}NO_6$	756.5198	0.0076	10.05	TAG 45:13	$[M+NH_4]^+$
			$C_{41}H_{75}NO_9P$	756.5174	0.0100	13.22	PS 35:2	$[M+H-H_2O]^+$
			$C_{41}H_{75}NO_9P$	756.5174	0.0100	13.22	PAA 36:3	$[M+H]^+$
			$C_{40}H_{79}NO_9K$	756.5386	-0.0112	-14.80	HexCer 34:0;O3	$[M+K]^+$
**775.5374**	775.5193 (DHA)	775.5063 (DHA)	$C_{40}H_{76}N_2O_{10}P$	775.5232	-0.0039	-5.03	PS 34:3	$[M+NH_4]^+$
			$C_{43}H_{77}O_8PNa$	775.5248	-0.0055	-7.09	PA 40:4	$[M+Na]^+$
			$C_{40}H_{81}O_9PK$	775.5250	-0.0057	-7.35	PG O-34:0	$[M+K]^+$
			$C_{41}H_{76}O_{11}P$	775.5120	0.0073	9.41	PI O-32:2	$[M+H-H_2O]^+$
			$C_{45}H_{76}O_8P$	775.5272	-0.0079	-10.19	PA 42:7	$[M+H]^+$

* As displayed in Fig. 5.

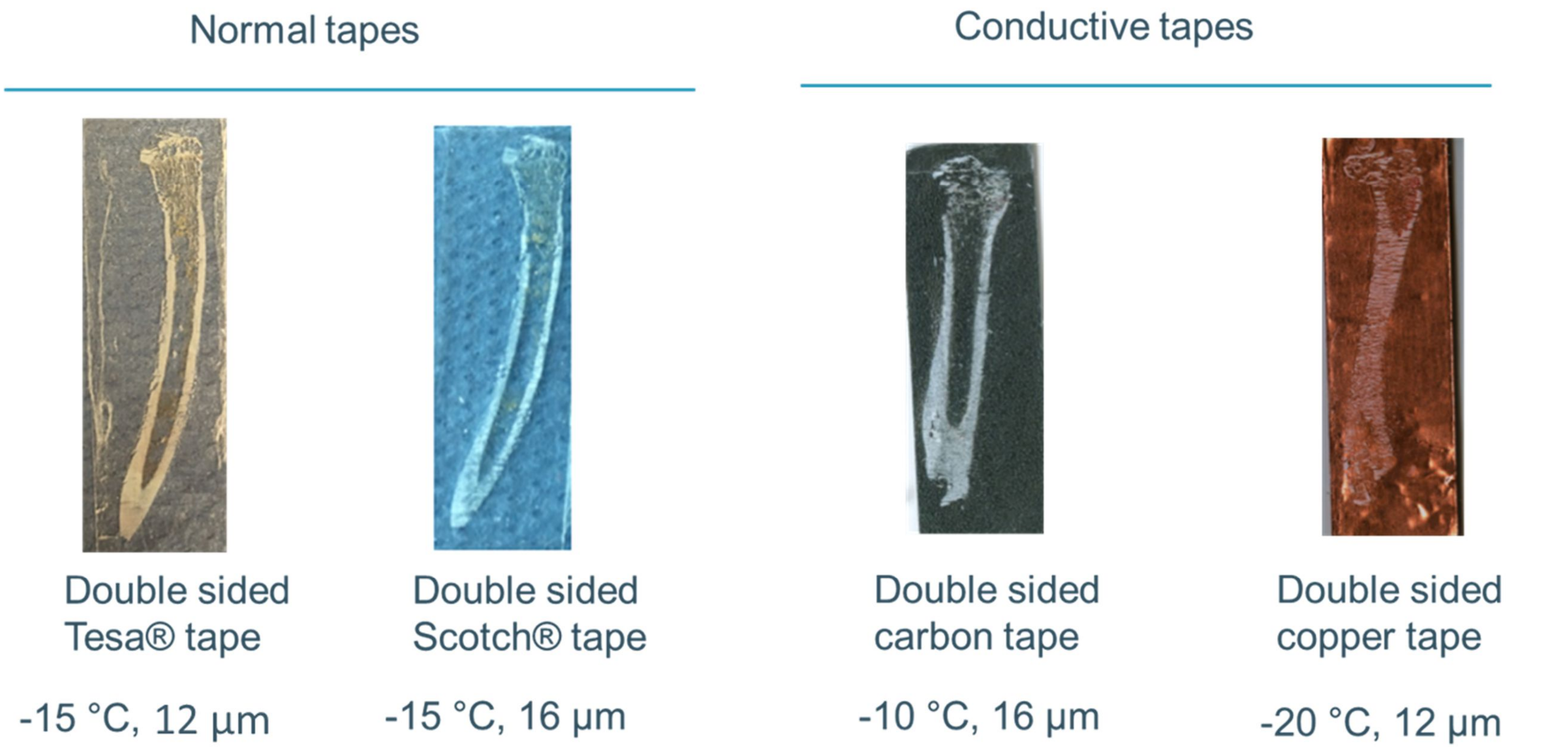

Fig. S1 Optical images of rat bones mounted on each of the four tapes used during sectioning with optimized section thickness and temperature

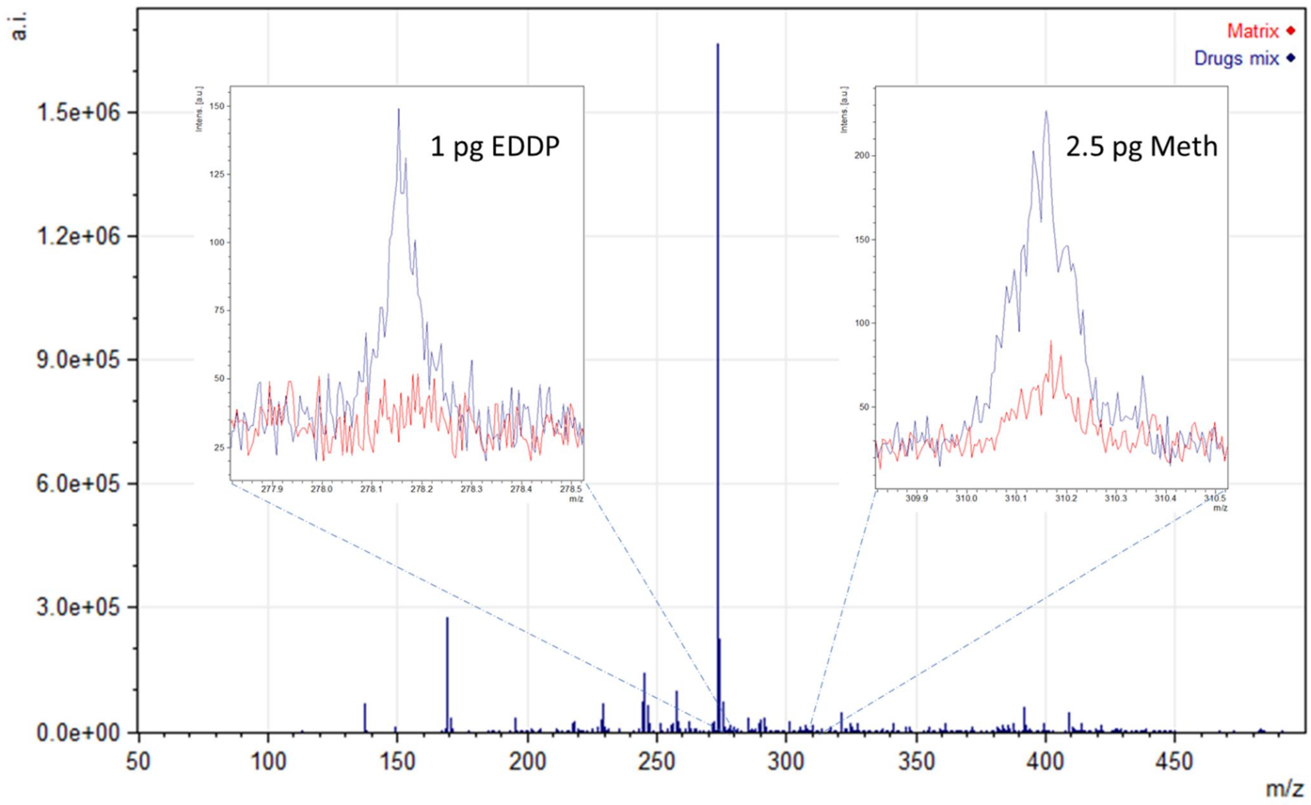

Fig. S2 Using DHB as matrix in positive ion mode. In blue the mass spectrum of the measured droplet with a drug mixture of 1pg EDDP (*m/z* 278.18) and 2.5 pg methadone (*m/z* 310.21) and in red the spectrum of DHB

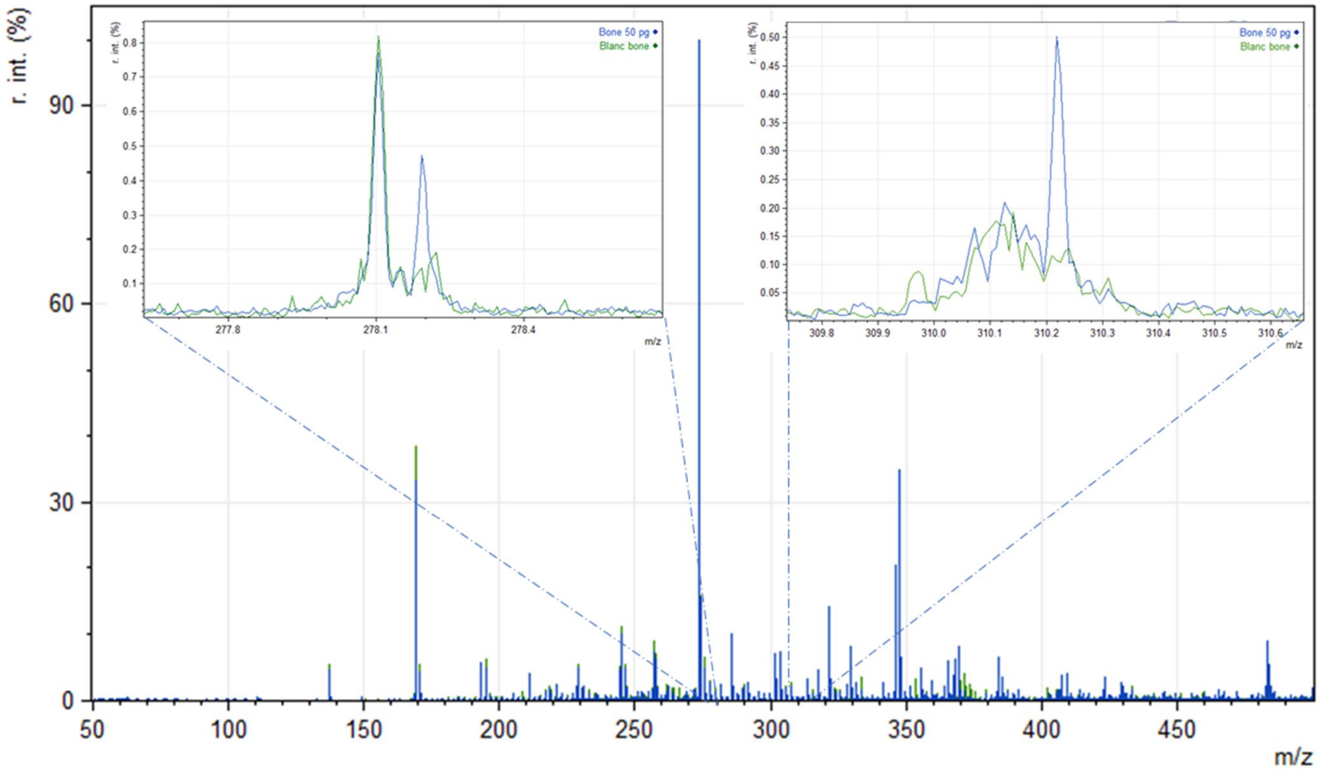

Fig. S3 Using DHB as matrix in positive ion mode. In blue the mass spectrum of a spot with 50 pg methadone and 50 pg EDDP on bone tissue and in green a mass spectrum of blanc bone tissue

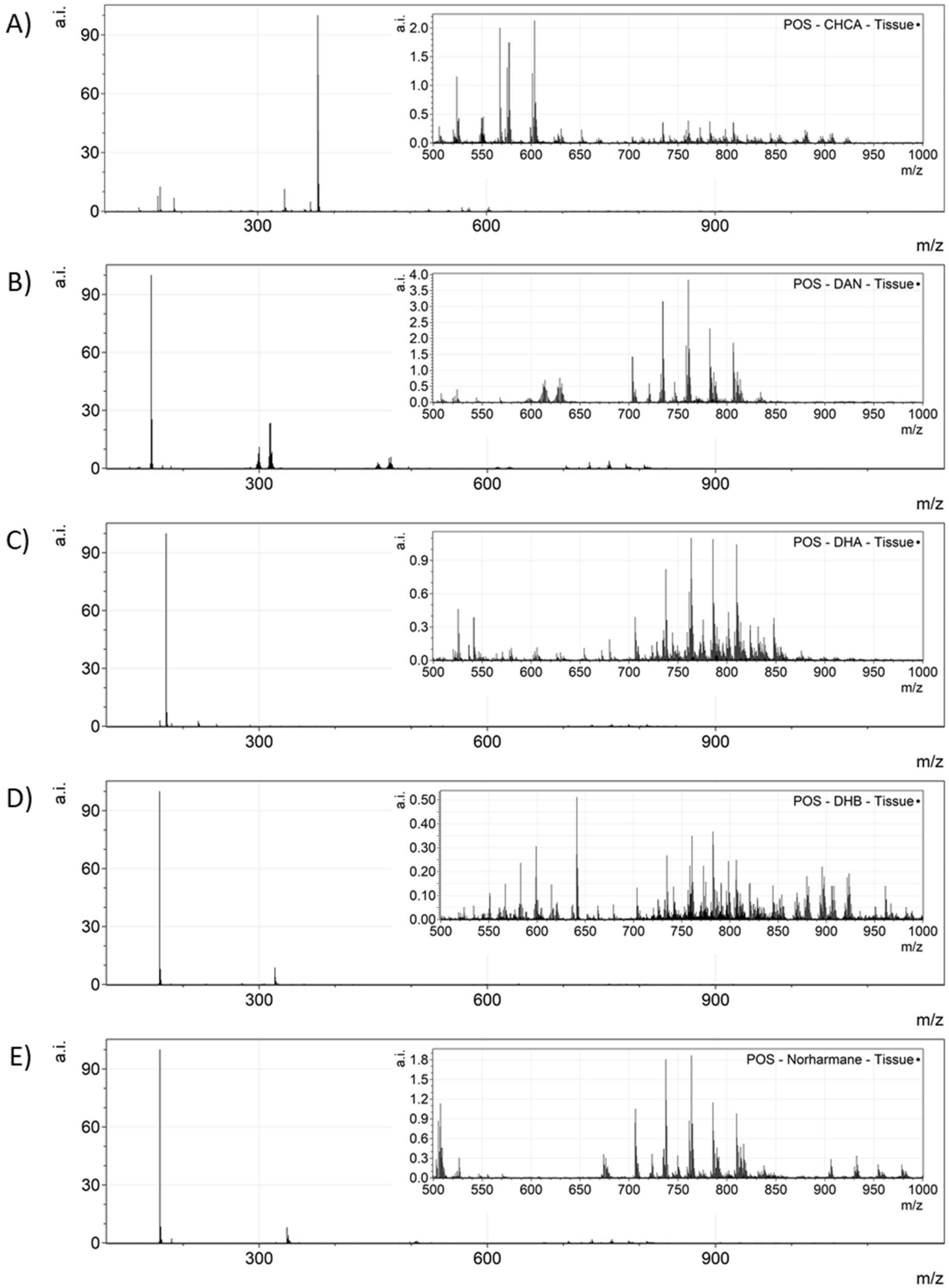

Fig. S4 Profile mass spectra of the measured area for the different matrices in positive ion mode using mice bone for the untargeted application. Full range (*m/z* 100-1200) mass spectra with an insertion of a zoom in of the *m/*z range 500-1000 for CHCA (A), DAN(B), DHA (C), DHB (D), and norharmane (E). Mass spectra represent the combined signal from bone, bone marrow, surrounding (muscle) tissue and some surrounding background signal

Information S1: Untargeted application for lipids in negative ion mode using mice hind legs

For the selection of matrices to test for their suitability for the detection of lipids in bone and bone marrow in negative ion mode, DAN, NEDC, and norharmane were compared. NEDC was included in this analysis although it is more often used for the detection of metabolites than for lipids, as multiple molecules in the lower mass range were seen during initial analysis.

In negative ion mode, different matrices allowed for the detection of different ions from the bone and bone marrow and most *m/z* values were only presented in one measurement (see Fig. S5). For DAN and norharmane, it was only possible to obtain specific signals for bone marrow, while for NEDC, it was possible to observe specific signals from both, bone and bone marrow. NEDC had the highest number of *m/z* values specific for bone or bone marrow, of which most of the *m/z* values were obtained from the bone (see Table S4). However, these specific *m/z* values are only in the lower mass range (< *m/z* 500). DAN resulted in the highest number of specific *m/z* values for bone marrow. Furthermore, NEDC has higher signal intensities for bone and relatively high S/N values for bone marrow (see Table S4), while the intensities and S/N values for bone marrow are higher in DAN and norharmane. DAN showed the highest S/N values for bone marrow. In negative ion mode, DAN and NEDC have the most interference of matrix peaks with the tissue-related peaks, while there is some interference for norharmane (see mass spectra in Fig. S6).

Of the compared matrices in negative ion mode, DAN caused an unwanted reaction with the double-sided tape. For the matrices tested in negative ion mode, it was possible to create distribution images up to *m/z* 500 (see Fig. S5). For DAN and norharmane, a few specific *m/z* values for bone marrow could be found above *m/z* 500, but not for NEDC. With limited or no detection of lipids in bone and bone marrow, major lipid classes can be missed, for example, certain subclasses phospholipids that ionize better in negative ion mode [6]. Although these subclasses have been shown to play a role in bone health [7]. No specific *m/z* values for bone tissue could be obtained with DAN and norharmane. Based on specific *m/z* values, NEDC is the only matrix for which specific *m/z* values for bone as well as bone marrow could be obtained. However, the background signal and interference of this matrix is high (see Fig. S6). In negative ion mode, none of the tested matrices provided the desired result in terms of detection of *m/z* values specific for bone and bone marrow in combination with low interference of the matrix with the signal from the tissue. Therefore, further testing of the best matrix in negative ion mode is necessary.

Table S4 Comparison of DAN, NEDC, and norharmane in negative ion mode using mice hind legs for the untargeted application. The number of specific *m/z* values is given for bone as well as bone marrow, which are the number of specific *m/z* values from the 2000 most intense peaks as selected by HDImaging (version 1.4, Waters Corporation). From these specific *m/z* values, values potentially related to the matrix and background were removed by comparing them to a measurement of the background. The average signal-to-noise (S/N) is calculated for the specific *m/z* values and the ratio to the background is calculated in comparison to the highest background peak, based on the S/N-values obtained after peak picking using mMass (Open Source Mass Spectrometry Tool, version 5.5.0). In addition, the interference of the matrix with signal from the tissue is given based on the average mass spectra looking at the amount of background signal and the overlap of background peaks with peak specific for tissue

**Matrix**	**Specific *m/z* values**			**Signal-to-noise (S/N) ratio**				**Interference of matrix with tissue signal**
	Bone	Bone marrow	Total	Average bone	Ratio to background (%)	Average bone marrow	Ratio to background (%)	
**DAN**	0	14	14	-	-	17186.58	0.63	Middle
**NEDC**	20	6	26	25773.37	4.61	2990.28	0.54	Extremely high
**Norharmane**	0	9	9	-	-	5846.46	0.02	Middle

Table S5: Tentative identifications of selected *m/z* values in negative ion mode. Per *m/z* value from the merged dataset, the experimental *m/z* value from the average mass spectrum of the corresponding matrix, the *m/z* value for a random pixel (from the corresponding matrix), the matched molecular formula, the theoretical *m/z* value, the Δm ($m/z_{exp} - m/z_{theo}$), the ppm error ($\frac{m/z_{exp} - m/z_{theo}}{m/z_{theo}} \times 10^6$), the potential matching lipid(s), and the detected ion are provided

Abbreviations lipid classes: CAR = acyl carnitine; FA= fatty acids; LPA = lyso-glycerophosphate; LSM = lyso-sphingomyelin; NAE = N-acyl ethanolamines; ST = sterols

Merged dataset *m/z* value*	Experimental *m/z* value average spectrum (matrix)	*m/z* value random pixel (matrix)	Molecular Formula	Theoretical *m/z*	Δm	Ppm error	Possible lipid	Ion
**259.1009**	259.0982 (NEDC)	259.0036 (NEDC)	$C_{15}H_{15}O_4$	259.0976	0.0006	2.32	FA 14:7	$[M+HCOO]^-$
**283.2673**	283.2640 (DAN)	283.2640 (DAN)	$C_{18}H_{35}O_2$	283.2643	-0.0003	-1.06	FA 18:0	$[M-H]^-$
**436.3118**	436.3096 (DAN)	436.3124 (DAN)	$C_{25}H_{42}NO_5$	436.3069	0.0027	6.19	CAR 18:3;O	$[M-H]^-$
			$C_{25}H_{42}NO_5$	436.3069	0.0027	6.19	NAE 21:4;O	$[M+CH_3COO]^-$
			$C_{25}H_{42}NO_5$	436.3069	0.0027	6.19	NAE 22:4;O	$[M+HCOO]^-$
			$C_{25}H_{42}NO_5$	436.3069	0.0027	6.19	NAE 21:4	$[M+CH_3OCOO]^-$
**437.3100**	437.3100 (Norharmane)	437.3044 (Norharmane)	$C_{29}H_{41}O_3$	437.3061	0.0039	8.92	ST 27:5;O	$[M+CH_3COO]^-$
			$C_{21}H_{46}N_2O_5P$	437.3150	-0.0050	-11.39	LSM 16:0;O2	$[M-H]^-$
			$C_{22}H_{46}O_6P$	437.3038	0.0063	14.29	LPA O-19:0	$[M-H]^-$

* As displayed in Fig. S5.

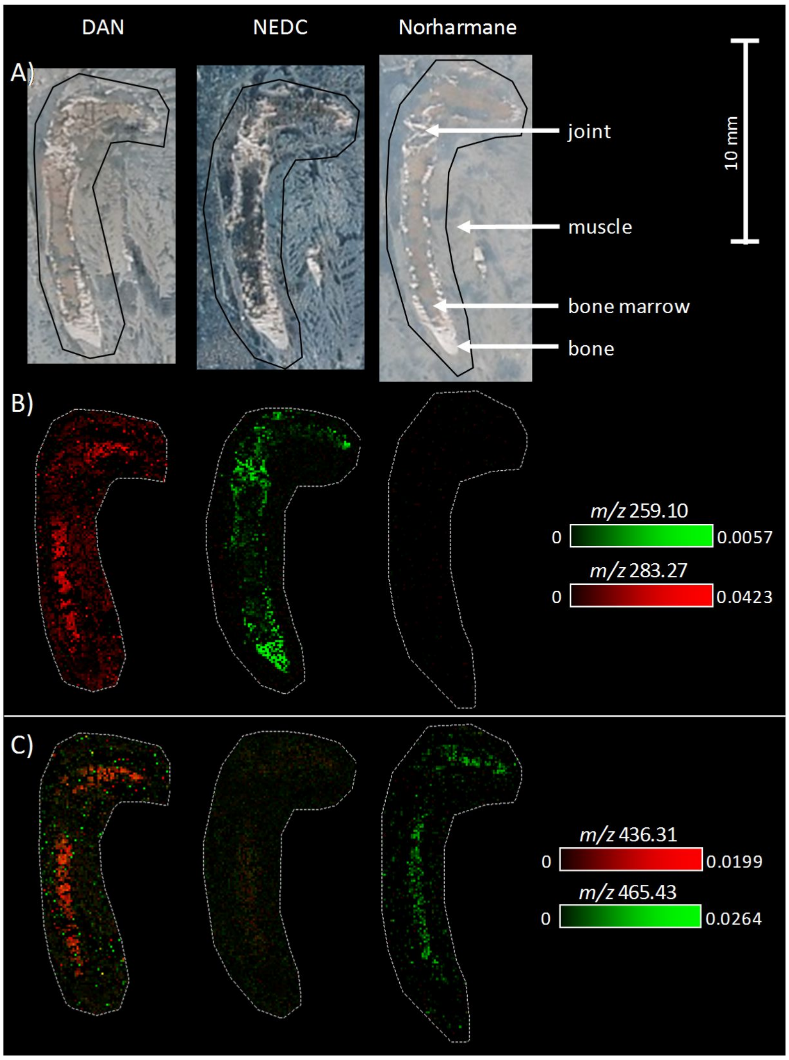

Fig. S5 Overlay images of selected *m/z* values for DAN, NEDC, and Norharmane in negative ion mode measured with MALDI-MSI of mice hind legs for the untargeted detection of lipids. The m/z values were selected to show the agreement and differences between the matrices in terms of desorption and ionization efficiency. A) Optical scan of the section with the measured area indicated with a black line. Overlay images of the distributions of selected *m/z* values in the range 200-300 (B), and 300-500 (C). The distribution images are total ion current (TIC) normalized and the intensity scale show the maximum relative intensity of the specific *m/z* value. For tentative identifications of the selected *m/z* values see Table S5

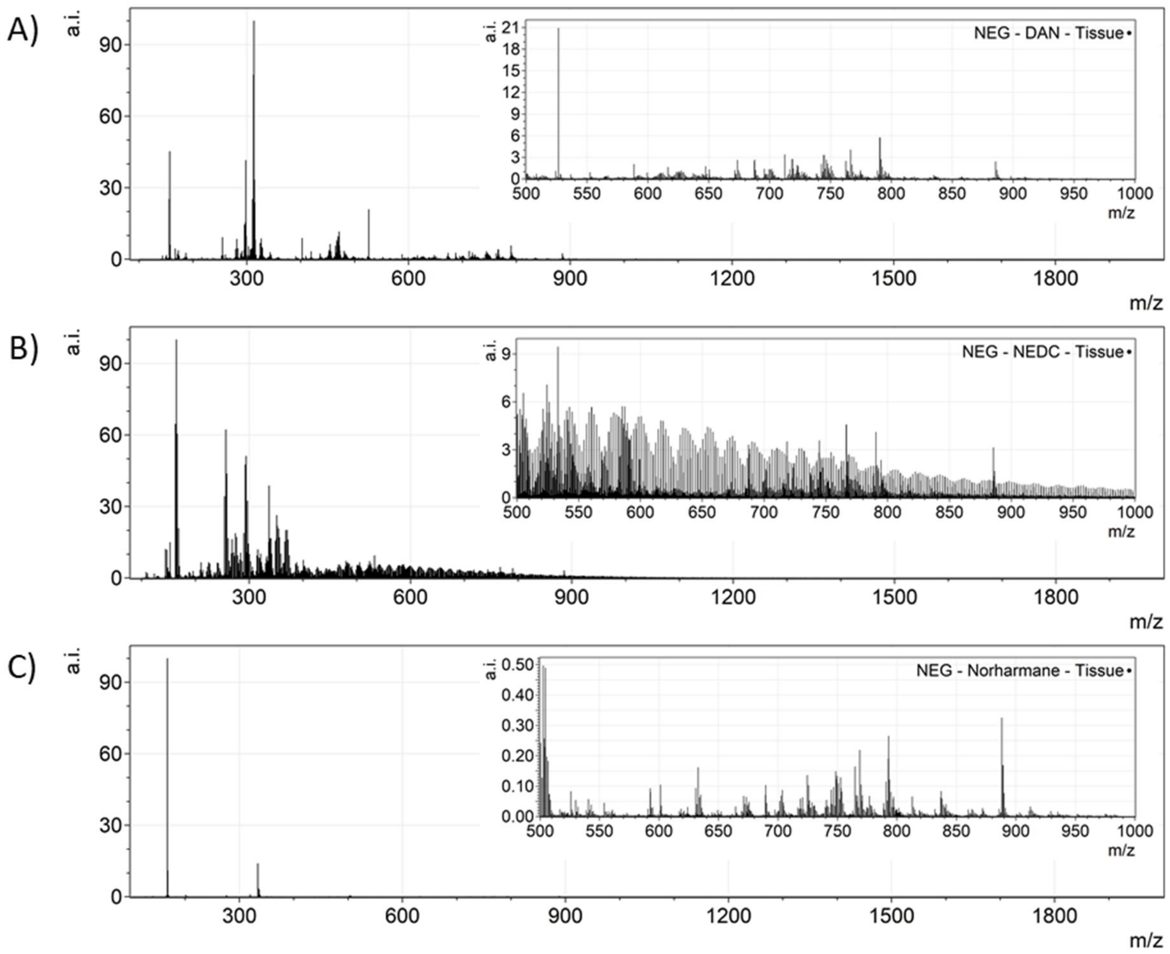

Fig. S6 Profile mass spectra of the measured area for the different matrices in negative ion mode using mice bone for the untargeted application. Full range (*m/z* 100-2000) mass spectra with an insertion of a zoom in of the *m/*z range 500-1000 for DAN (A), NEDC (B), and norharmane (C). Mass spectra represent the combined signal from bone, bone marrow, surrounding (muscle) tissue and some surrounding background signal

References

1. Seeley EH, Wilson KJ, Yankeelov TE, Johnson RW, Gore JC, Caprioli RM, et al. Co-registration of multi-modality imaging allows for comprehensive analysis of tumor-induced bone disease. Bone [Internet]. 2014/01/30. 2014 Apr;61:208–16. Available from: https://www.ncbi.nlm.nih.gov/pubmed/24487126
2. Fujino Y, Minamizaki T, Yoshioka H, Okada M, Yoshiko Y. Imaging and mapping of mouse bone using MALDI-imaging mass spectrometry. Bone Reports [Internet]. 2016 Dec;5:280–5. Available from: http://dx.doi.org/10.1016/j.bonr.2016.09.004
3. Schaepe K, Bhandari DR, Werner J, Henss A, Pirkl A, Kleine-Boymann M, et al. Imaging of Lipids in Native Human Bone Sections Using TOF–Secondary Ion Mass Spectrometry, Atmospheric Pressure Scanning Microprobe Matrix-Assisted Laser Desorption/Ionization Orbitrap Mass Spectrometry, and Orbitrap–Secondary Ion Mass Spectrometry. Anal Chem [Internet]. 2018 Aug 7;90(15):8856–64. Available from: https://doi.org/10.1021/acs.analchem.8b00892
4. Svirkova A, Turyanskaya A, Perneczky L, Streli C, Marchetti-Deschmann M. Multimodal imaging of undecalcified tissue sections by MALDI MS and μXRF. Analyst [Internet]. 2018;143(11):2587–95. Available from: http://dx.doi.org/10.1039/C8AN00313K
5. Muddiman DC, Khodjaniyazova S, Hanne NJ, Cole JH. Mass spectrometry imaging (MSI) of fresh bones using infrared matrix-assisted laser desorption electrospray ionization (IR-MALDESI). Anal Methods. 2019;11(46):5929–39.
6. Fernandez J, Ochoa B, Fresnedo O, Giralt MT. Matrix-assisted laser desorption ionization imaging mass spectrometry in lipidomics. Anal Bioanal Chem. 2011;401:29–51.
7. During A, Penel G, Hardouin P. Progress in Lipid Research Understanding the local actions of lipids in bone physiology. Prog Lipid Res [Internet]. 2015;59:126–46. Available from: http://dx.doi.org/10.1016/j.plipres.2015.06.002
